# Evaluation of Static Yield Stress and Buildability of PVA Fiber-Reinforced Mortars for 3D Printing Using a Vane Shear Test

**DOI:** 10.3390/ma19061093

**Published:** 2026-03-12

**Authors:** Shoma Uehara, Yusei Ohshiro, Kanako Shima, Kazuya Sakamoto, Kentaro Yasui

**Affiliations:** 1 Advanced Civil Engineering, National Institute of Technology, Kagoshima College, 1460-1 Shinko, Hayato-cho, Kirishima 899-5193, Kagoshima, Japan; 2 Technical Division, National Institute of Technology, Kagoshima College, 1460-1 Shinko, Hayato-cho, Kirishima 899-5193, Kagoshima, Japan; 3 General Technology Research Institute, Infratec Co., Ltd., 3141-1 Hiramatsu, Aira City 899-5652, Kagoshima, Japan; 4 Department of Urban Environmental Design and Engineering, National Institute of Technology, Kagoshima College, 1460-1 Shinko, Hayato-cho, Kirishima 899-5193, Kagoshima, Japan

**Keywords:** construction 3D printing, static yield stress, buildability, PVA fibers, maximum shear stress, 15-stroke flow, interlayer stability

## Abstract

**Highlights:**

- An assessment of early buildability and interlayer stability of 3D-printable mortars using the vane shear test.
- The correlation between the 15-stroke flow and vane shear test results.
- Key parameters for mixture design and construction management.
- Fundamental insights into the structural performance of printed elements.

**Abstract:**

Three-dimensional printing (3DP) has gained increasing attention in construction as a means of addressing labor shortages and improving efficiency. Various studies have investigated fiber-reinforced mortars for 3DP. However, only a few studies have examined mixture design strategies aimed at controlling early structural build-up, and the relationships between early structural build-up, printability, and interlayer stability remain largely unexplored. This study aimed to establish a practical method for evaluating the static yield stress and early buildability of 3DP mortars under construction-site conditions. Vane shear and 15-stroke flow tests were conducted to assess the static and dynamic behavior of mortars incorporating polyvinyl alcohol (PVA) fibers, and their compressive and flexural strengths were also evaluated. According to the results, the vane shear test sensitively captured the rheological changes associated with variations in fiber content and superplasticizer dosage. The addition of PVA fibers increased the maximum shear stress of the mortar, resulting in atypical static yield stress development compared to fiber-free mortars. While the 15-stroke flow test further elucidated flowability, the vane shear test revealed a stronger correlation between mechanical properties and overall buildability. Thus, vane shear testing can be reliably used to assess early-age structural build-up and interlayer stability in 3DP mortars for optimizing print performance.

## 1. Introduction

In recent years, the construction industry has faced various challenges associated with labor shortages [1,2]. Among the potential solutions, three-dimensional printing (3DP) has attracted increasing attention for its ability to improve productivity and reduce environmental impacts [3,4,5]. In addition, various applications have been reported worldwide—from exterior repair walls to low-cost housing and bridge components—demonstrating the rapid advancements and diversification of this technology [6,7,8,9,10,11].

In Japan, however, the architectural applications of 3DP remain limited to small-scale structures owing to strict regulations under the Building Standards Act [12]. In civil engineering, 3DP has shown promising results, such as precast coastal blocks with abrasion resistance comparable to high-strength concrete [13] and bridge-pier prototypes exhibiting high dimensional accuracy [14]. In addition, several committees, including those established by the Japan Concrete Institute, the Architectural Institute of Japan, and the Japan Society of Civil Engineers, have been promoting standardization and technological development [1,15]. Nevertheless, the domestic implementation of 3DP is still lagging behind global trends.

**Figure 1:**
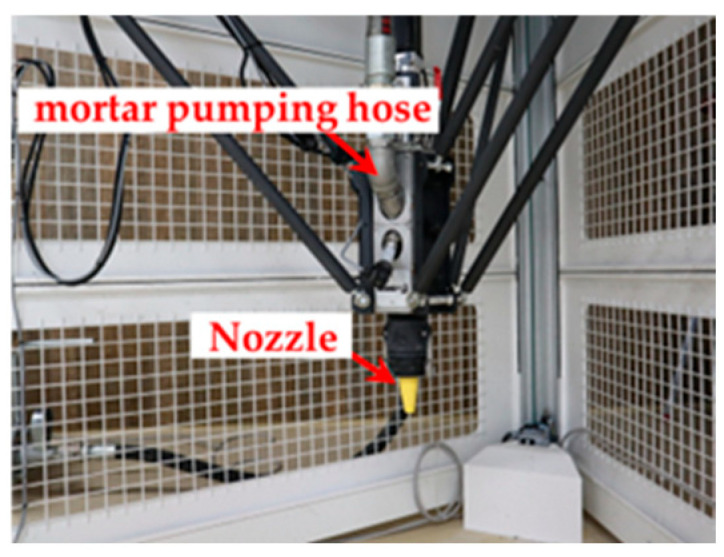
Construction 3D printer.

Material-extrusion 3DP (Figure 1) offers design flexibility and reduced construction time by enabling fabrication without formwork [1,3,7,16,17,18]. Mortars used in this method must simultaneously ensure extrudability, stable discharge, and interlayer stability, all of which are strongly influenced by time-dependent changes in static yield stress and shear-induced structural breakdown and rebuilding [17,18,19,20,21]. Such materials typically exhibit a decrease in viscosity under shear and a recovery of their internal structure once the shear is removed [21,22,23,24,25,26,27]. Because 3DP mortars rely on highly specialized compositions, traditional quality-control methods are often unsuitable [3], highlighting the need for simple, field-oriented evaluation techniques.

Previous studies have evaluated time-dependent changes in material structure and viscosity using rotational viscometers [18] or field-oriented methods, such as cone penetration, extrusion, and hardness tests [28,29,30]. However, these approaches often require complex equipment or lack sensitivity [31]. To address these challenges, the present study focuses on evaluating static yield stress and early structural build-up using the vane shear test, which provides a straightforward means of calculating maximum shear stress under field conditions [30,32,33,34].

**Figure 2:**
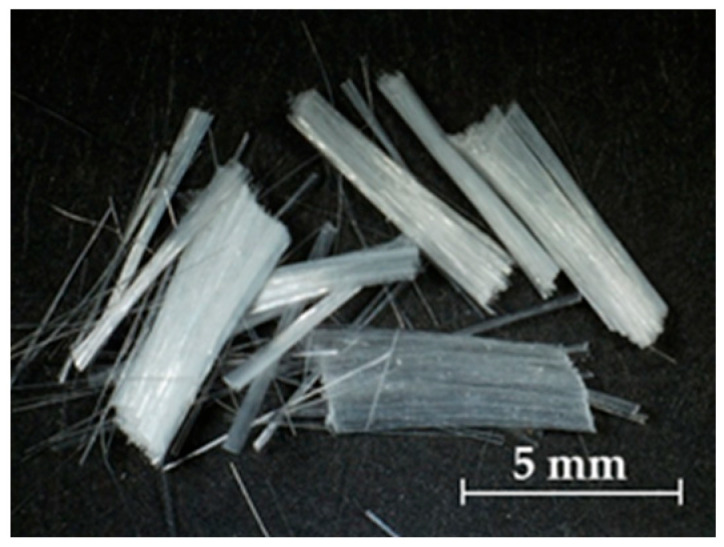
PVA fibers.

Recent studies have confirmed that incorporating fiber reinforcement into 3DP mortars enhances shape retention and interlayer adhesion during deposition [35]. Subsequent studies investigating various fiber types—including polyvinyl alcohol (PVA) (Figure 2) [35,36,37], basalt [38], polypropylene [39], natural fibers [27,40], and continuous fibers such as carbon and steel [7]—highlight their effects on hardening characteristics, bridging behavior, and toughness. However, the influence of fiber addition on early structural build-up and interlayer stability remains insufficiently quantified, and many existing mixtures lack detailed formulation data necessary for reproducibility.

In this study, we quantitatively evaluated the influence of PVA fiber addition on static yield stress and structural build-up using vane shear and 15-stroke flow methods. The relationships between mixture design, early structural build-up, and mechanical performance were also investigated. To this end, both the time-dependent compressive and flexural strengths and the as-printed geometries were obtained from a layering experiment employing an actual 3D printer.

The findings of this study provide insights into mixture design and construction management for 3DP mortars and clarify their relationship with interlayer stability.

The remainder of this paper is organized as follows. Section 2 describes the 3DP mortar preparation, static yield stress evaluation methods, and mechanical characterization tests. Section 3 compares the results obtained from the vane shear and 15-stroke flow tests, discusses high-sensitivity procedures for detecting changes in static yield stress and early structural build-up, and further examines the mechanical properties of mortar. Section 4 summarizes the findings and outlines future research.

## 2. Experiment Overview

### 2.1. Materials and Specimen Preparation

The materials used in this study are listed in Table 1. Blast furnace slag cement Type B (BB), containing 40–45% blast-furnace slag, and silica fume (SF) were employed as binders (B). Finely ground calcium carbonate (CaCO_3_, denoted as Ca) was added to improve the flowability of the mortar and enhance its early strength development. The use of SF, with a particle size smaller than cement, is expected to improve workability by filling the voids between cement particles and densifying the matrix [41]. Silica sand (S) (particle size: 0.212–1.18 mm) was used as the fine aggregate. A naphthalene-based retarding superplasticizer (SP) served as the chemical admixture. The water-reducing mechanism of naphthalene-based admixtures relies solely on electrostatic repulsion generated by sulfonic acid groups. Consequently, their adsorption is less affected by alkali components leached from cement compared to that of polycarboxylate-based admixtures, which are highly sensitive to such factors. This results in superior material versatility. Moreover, despite the growing use of alternative powders, such as calcium carbonate and blast furnace slag, to reduce cement content, naphthalene-based admixtures remain stable and effective [42].

PVA fibers (F) (length: 6 mm; diameter: 40 μm) were used as reinforcement. This fiber length, as established previously [43], prevents clogging in a 20-mm-diameter printhead. PVA contains numerous hydroxyl groups along its polymer chain, which act as hydrophilic sites and promote strong chemical bonding with the cement paste [39,44,45,46,47]. In addition, alkali resistance allows PVA fibers to remain stable in mortar, limiting microcracking and inhibiting long-term toughness degradation through crack bridging [46,47].

The mix proportions of the 3DP mortars are listed in Table 2. The cement content was set high to impart sufficient viscosity and ensure dimensional stability after layering during extrusion–based 3DP. The sand-to-powder ratio (S/P) was defined as the ratio of fine aggregate to the combined content of BB, SF, and Ca. The binder-to-sand ratio (B/S) was selected carefully to: (1) balance extrusion stability and post-layering shape retention, (2) optimize the extrusion performance of the printer, and (3) prevent layer deformation. The SP dosages were set at 0.9% (SP9) and 1.1% (SP11) by mass of mixing water and were internally adjusted within the total water content. This allowed for comparison of the effects on flow behavior and early-age rheology due to improved flowability and delayed initial setting. PVA fiber dosages were set at 0%, 0.3%, and 0.6% by volume of mortar and were externally added to examine the influence of fiber addition on toughness and potential fiber agglomeration.

Additionally, the mortars marked with asterisks (*) in Table 2 were used in the layering experiments. In these experiments, two mixtures were prepared and subjected to layering tests to clarify the influence of fiber addition on interlayer stability and mechanical performance: one mixture contained 0.3% PVA fibers, whereas the other, used for comparison, was an unsuitable mix lacking sufficient interlayer stability.

The mortar was prepared using a 10 L vibrating mixer (OM-10E, Tiger Chiyoda Materials Co., Ltd., Yokohama, Japan). BB, SF, Ca, and S were first dry-mixed at 400 rpm for 1 min. Then, water containing SP9 or SP11 was added, and mixing was continued for 5 min. Subsequent addition of PVA fibers was followed by an additional 1 min of mixing to ensure homogeneous dispersion of all materials.

For strength testing, the mortar was cast into cylindrical molds (diameter: 50 mm; height: 100 mm) and prismatic molds (40 mm × 40 mm × 160 mm). The specimens were compacted using a table vibrator (TS-450×600, Mikasa Sangyo Co., Ltd., Tokyo, Japan), sealed, and cured in a constant-temperature chamber at 20 °C for 1, 3, 7, 28, and 56 days.

### 2.2. Test Methods

(1) Vane shear test

**Figure 3:**
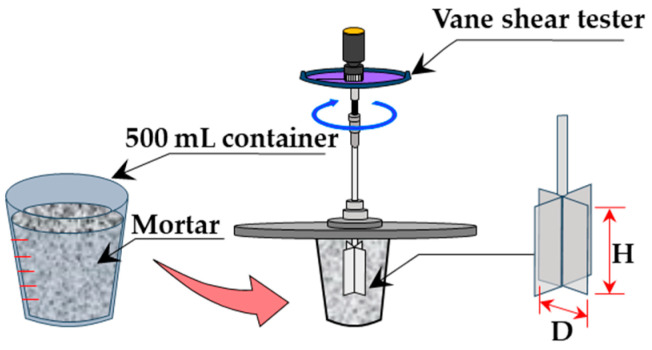
Vane shear test in progress.

The vane shear test procedure is shown in Figure 3, and the specifications of each vane blade are listed in Table 3. In this test, a vane blade was inserted in container into a 500-mL container containing freshly mixed mortar and rotated at a constant rate of 10°/s. The maximum resistance torque *M_max_* (cN·m) was measured to calculate the maximum shear stress *τ_v_* (kN/m^2^) using a simplified form of Cadling’s equation [33,48] as follows:

*τ_v_* = *M_max_*/π(*D*^2^*H*/2 + *D*^3^/6), (1)

where *D* is the vane blade diameter (mm), and *H* is the vane blade height (mm). To prevent interference from the failure zone generated by the vane blade during testing, separate mortar samples were prepared for each measurement immediately after mixing.

(2) 15-stroke flow test

**Figure 4:**
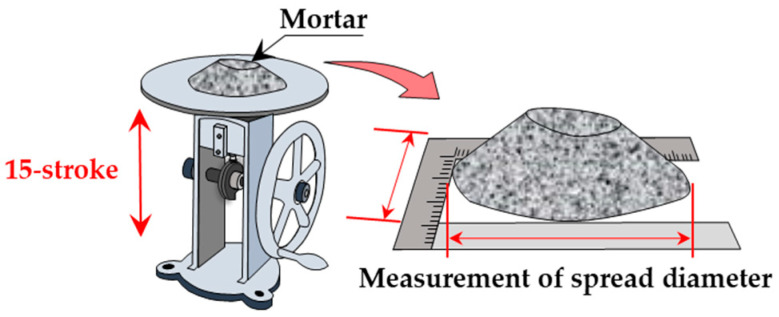
15-stroke flow test in progress.

The 15-stroke flow test (Figure 4) was conducted in accordance with JIS R 5201 [34]. The flow cone (upper diameter: 70.0 ± 0.5 mm; lower diameter: 100.0 ± 0.5 mm; height: 60.0 ± 0.5 mm) was filled with mortar and lifted vertically; the flow table was dropped 15 times, and the resulting spread diameter was measured to evaluate the material’s resistance to shear-induced deformation. For all mortar mixes, the initial flow value (at zero drops) was 100 mm. Therefore, a smaller spread after 15 strokes indicated higher shear resistance.

The vane shear and 15-stroke flow tests were conducted 0, 5, 10, 15, 20, 25, and 30 min after mixing under the following two conditions:

- Static condition: After mixing (0 min), the mortar was left undisturbed until each test time. This condition simulates 3DP in which the material is extruded and deposited without external disturbance.
- Stirred condition: After mixing (0 min), the mortar was continuously stirred at 40 rpm until each test time. This condition simulates restirring for delayed setting when a significant time elapses before extrusion.

A similar approach has been previously used [49] to investigate the effect of resting and restirring on early-age material behavior via vane shear tests under different static and stirred conditions. In practical 3DP, materials may remain static for up to approximately 1 h after mixing before extrusion, or they may be restirred to delay setting. Thus, measuring both static and stirred conditions provides relevant information on flowability, extrusion stability, and interlayer adhesion, supporting the design of 3DP mortars.

(3) Compressive strength test

The compressive strength test was conducted in accordance with JIS R 1108 [50] at curing ages of 1, 3 (early strength), 7 (medium-term strength), 28 (standard strength), and 56 (long-term strength) days to comprehensively evaluate strength development over time.

(4) Flexural strength test

The flexural strength test followed JIS R 5201 [34], using the same testing ages as in the compressive strength test. These tests evaluated the effects of varying SP dosages and fiber contents on flexural strength.

Specimens for both compressive and flexural strength tests were prepared in two series: one from the laboratory experiments and another from the layering tests.

All tests were repeated three times under each condition, and the average of their results was reported as the final result.

(5) Printing experiment

**Figure 5:**
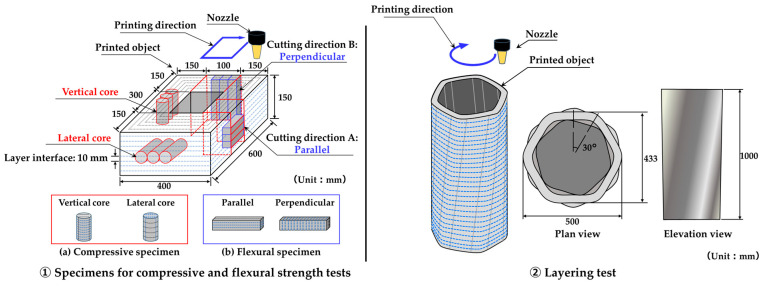
Design overview of the lamination experiment.

Figure 5 shows an overview of the laminated structure fabricated in this study and the locations where specimens were collected. Layering tests were conducted using a material extrusion-type 3D printer (Delta WASP 3MT CONCRETE, WASP Ltd., Massa Lombarda, Italy) (Figure 1). During layering, the mortar discharge rate, layer interface width, column interface width, and nozzle travel speed (layer path speed) were set at 1.2 L/min, 10 mm, 20 mm, and 100 mm/s, respectively.

Nozzle diameters in the range of 15–30 mm have been widely used and studied with respect to extrusion stability and strand geometry, supporting the representativeness of the 20-mm diameter adopted here [51]. The layer height of 10 mm is less than half of the nozzle diameter, which is consistent with common practice to maintain strand stability and buildability [52]. Printing speeds in similar systems generally range from 50–200 mm/s, placing the 100 mm/s used in this study within a commonly applied interval [53] and also matching the operating speed recommended by the manufacturer. The discharge rate (~1.2 L/min) was also consistent with the volumetric flow rate calculated from the strand width (20 mm), layer height (10 mm), and nozzle travel speed (100 mm/s). These considerations indicate that the printing conditions employed in this study fall within typical extrusion-based 3D concrete printing operating parameters.

Interlayer stability refers to the ability to firmly stack layers without deformation or collapse [54]. Its evaluation was based on the maximum shear stress (described later), and feasibility was determined from differences in these values.

For mechanical testing, specimens were extracted from the printed elements (Figure 5). Compressive strength specimens were prepared in accordance with JSCE-F 711-2025 [4]. Core samples were taken in two orientations: (a) vertical and (b) lateral. Three cores (diameter: 45 mm; length: 150 mm) were extracted per orientation.

For all specimens, the targeted interlayer spacing (10 mm) was kept constant across the cutting path, interface planes were centered within the prism depth, and edge or end regions affected by startup or termination effects were excluded. This approach improves reproducibility and enables direct comparison of anisotropic responses. To avoid the influence of surface irregularities formed during printing, cores were taken from interior regions of the printed elements at positions with uniform cross-sections, excluding the ends.

Each core was ground to a height of 90 mm, and compressive strength tests were conducted after 3, 7, 14, and 28 days of curing. In contrast, flexural strength specimens were obtained by cutting the printed elements used in the layering tests in both vertical and horizontal directions relative to the interlayer interfaces. These were subsequently shaped into specimens measuring 40 mm × 40 mm × 160 mm.

Together, these tests enabled a multifaceted evaluation of the overall mechanical performance of laminated structures, accounting for the effects of fiber orientation, anisotropy arising from the layering process, and interlaminar adhesion.

## 3. Results and Discussion

### 3.1. Vane Shear Test

**Figure 6:**
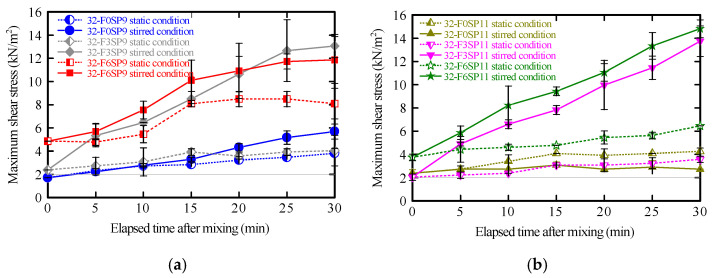
Time-dependence of maximum shear stress. (**a**) Effect of fiber content, 0.9% superplasticizer (SP). (**b**) Effect of fiber content, 1.1% SP.

The time-dependent changes in the maximum shear stress of the mortar under static and stirred conditions are shown in Figure 6. For all mixtures, the maximum shear stress increased with time after mixing as hydration progressed. Although the initial values differed depending on the fiber and SP contents, no significant difference was observed in the overall rate of stress increase.

In materials exhibiting structural build-up, agitation typically disrupts early hydration products, which temporarily suppresses hydration and maintains flowability; therefore, the increase in maximum shear stress is expected to be small [55]. However, in this study, the stirred condition exhibited higher maximum shear stress than the static condition. This atypical behavior is attributed to the hydrophilic PVA fibers, which absorb water and develop bridging interactions within the cement paste, thereby reinforcing the internal structure and increasing shear resistance [35,36,37].

The vane shear test does not directly measure viscosity or dynamic yield stress; instead, the maximum shear stress obtained from the test reflects the static yield stress required to initiate flow. Monitoring its time-dependent evolution enables a semi-quantitative assessment of structural build-up and shear resistance in the mortar [56]. Notably, high-shear extrusion behavior and viscosity recovery are beyond the scope of this method.

In this study, the maximum shear stress was adopted as a practical indicator for 3DP operations. Higher values—particularly under stirred conditions—indicated that fiber addition promoted structural reinforcement and reconstruction, improving buildability and interlayer stability. When the lower control limit was set at 2 kN/m^2^, all mixtures exceeded this threshold.

Overall, PVA fiber addition increased the internal structural integrity of the mortar, leading to higher static yield stress. Although the vane shear test is less comprehensive than rheometer-based measurements, it remains a robust, practical, and effective method for evaluating the early-age stability of 3DP mortars.

### 3.2. 15-Stroke Flow Test

**Figure 7:**
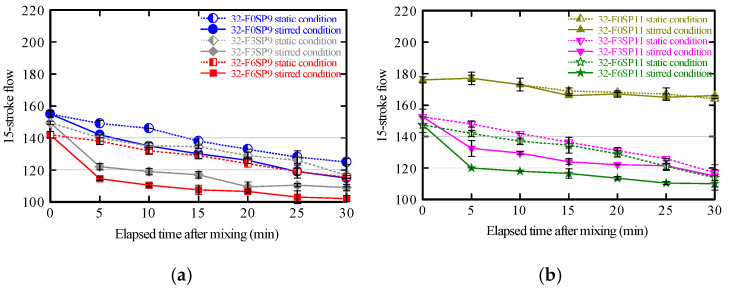
Time dependence of 15-stroke flow. (**a**) Effect of fiber content, 0.9% SP. (**b**) Effect of fiber content, 1.1% SP.

The time variations in the 15-stroke flow values under static and stirred conditions are shown in Figure 7. This widely recognized method for assessing the fresh properties of cementitious materials was used to evaluate the flowability and cohesiveness of the mortar.

**Figure 8:**
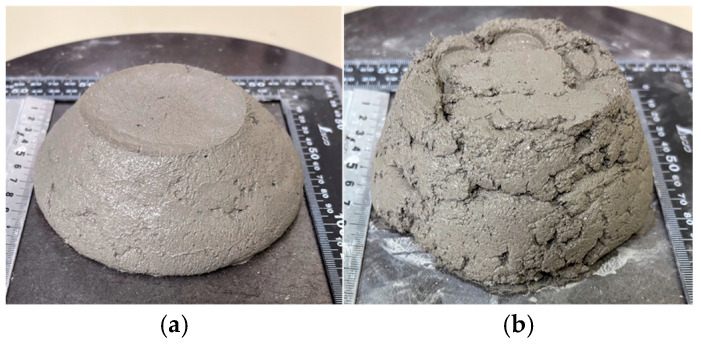
Appearance of mortar during 15-stroke flow test. (**a**) immediately after mixing. (**b**) after 30 min of mixing.

The flow values decreased with time after mixing, particularly under stirred conditions, indicating increasing difficulty in maintaining fluidity. This reduction is attributed to the redistribution and interaction of fibers during stirring, which can promote localized aggregation and hinder the resulting flow spread (Figure 8).

The SP improved initial fluidity in all mixtures; however, in fiber-reinforced mortars, fluidity retention decreased as setting progressed owing to their higher solid content and internal structural development. These findings confirm that maintaining flowability over time is more challenging in mixtures containing fibers.

Consequently, the 15-stroke flow test alone cannot quantitatively determine the shear yield stress. However, it offers useful supplemental information on time-dependent changes in flow resistance.

### 3.3. Relationship Between Maximum Shear Stress and 15-Stroke Flow

**Figure 9:**
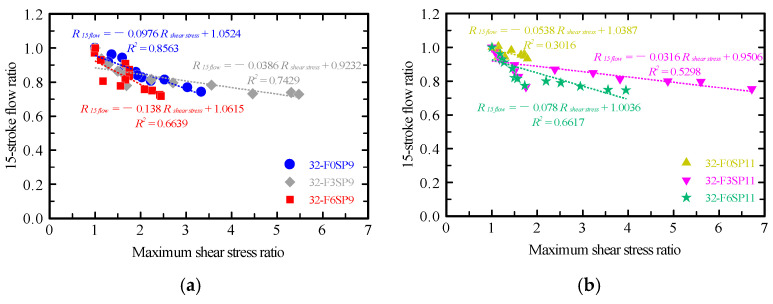
Relationship between maximum shear stress ratio and 15-stroke flow ratio. (**a**) Effect of fiber content, SP9. (**b**) Effect of fiber content, SP11.

The relationship between the maximum shear stress and 15-stroke flow ratios was obtained (Figure 9) to evaluate their correlation. Because the 15-stroke flow test alone has limitations in capturing time-dependent changes in fluidity, the results of the vane shear test were compared with those of this conventional method to clarify their relative positioning.

The shear stress ratio (*R_shear stress_*) represents the ratio of the maximum shear stress at each elapsed time to its initial value immediately after mixing (Figure 6). Similarly, the 15-stroke flow ratio (*R*_15 *flow*_) represents the ratio of the flow value at each elapsed time to the initial flow value (Figure 7).

For SP9 mixtures, a strong correlation (*R*^2^ = 0.86) was observed between the maximum shear stress and 15-stroke flow ratios in the fiber-free mortar. This indicates that changes in internal structure and decreases in flowability progressed almost proportionally. By contrast, for fiber contents of 0.3% and 0.6%, the coefficients of determination (*R*^2^) were 0.74 and 0.66, respectively, showing moderate correlations. Notably, the regression slope was steepest at the 0.6% fiber content, suggesting that changes in maximum shear stress were most pronounced relative to changes in the 15-stroke flow. This behavior is attributed to fiber aggregation and network formation within the mortar, which caused localized stress concentrations as the fiber content increased.

For SP11 mixtures, the fiber-free mortar exhibited a weak correlation (*R*^2^ = 0.30) between the maximum shear stress and 15-stroke flow ratios, indicating minimal to no relationship between them. This can be explained by the enhanced dispersion of binder particles resulting from the higher SP content, which diminishes the sensitivity of both shear stress and flow behavior. However, when fibers were added, the correlation reappeared (*R*^2^ = 0.66)—particularly at 0.6% fiber content. These results indicate that fiber aggregation and bridging promoted the development of a more integrated internal structure, simultaneously increasing shear stress and reducing flowability.

Thus, the R^2^ values, indicating the correlation between *R_shear stress_* and *R*_15 *flow*_, provide insight into the degree of flowability reduction and internal structural changes for each mixture. In other words, the flowability as well as the reconstruction of the internal structure induced by fibers can be quantitatively evaluated. In particular, when the 15-stroke flow ratio changed by 0.1, the maximum shear stress ratio varied by approximately 1. Thus, the vane shear test, which exhibits roughly ten times the sensitivity of the 15-stroke flow test, is an effective method for sensitively capturing internal structural changes in the mortar, and it contributes to assessing the self-supporting ability and internal structural reconstruction of mortars in 3DP.

### 3.4. Compressive Strength Test

**Figure 10:**
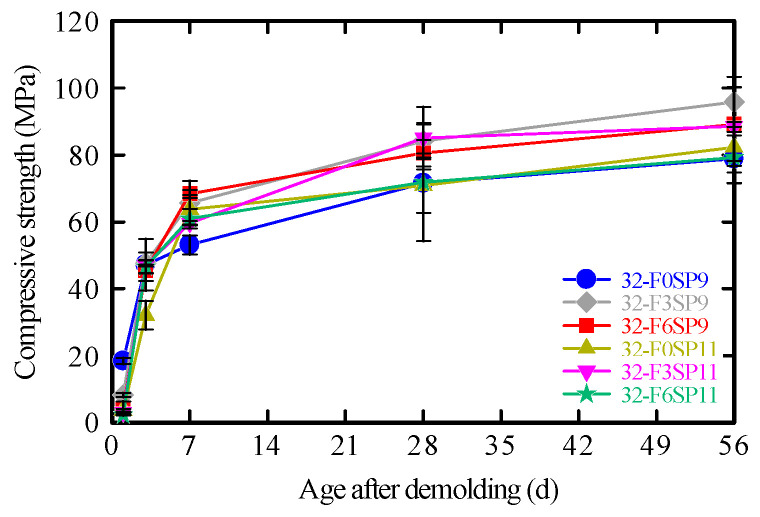
Compressive strength.

The compressive strengths of the 3DP mortars for each mix proportion are shown in Figure 10. For SP9 mixtures, the highest compressive strength was obtained at a fiber content of 0.3%. A similar trend was observed for the SP11 mixtures. The increase in strength owing to fiber addition can be attributed to the fibers restraining lateral deformation and delaying the initiation and propagation of microcracks under loading [43,44,57,58].

However, when the fiber content increased to 0.6%, the compressive strength decreased slightly compared to that at 0.3%. This reduction was likely due to the larger fiber volume replacing part of the cementitious matrix, which decreased the overall density and caused localized strength losses from fiber agglomeration [57].

Thus, while an appropriate fiber dosage enhances compressive strength, excessive fiber addition may lower mortar density and lead to poor fiber dispersion, thereby reducing strength. Furthermore, excessive use of SP can cause over-dispersion and bleeding [59], which may weaken the interfacial bonding between fibers and cement paste and suppress strength development.

### 3.5. Flexural Strength Test

**Figure 11:**
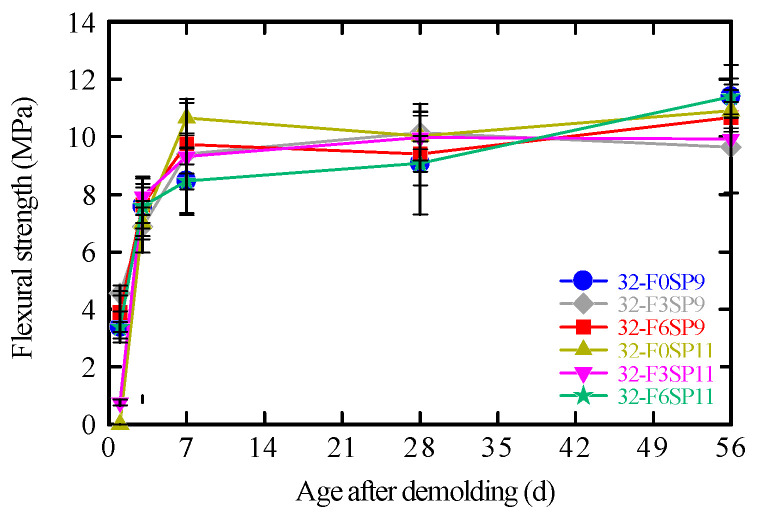
Flexural strength.

The flexural strengths of the 3D-printed mortars with different mix proportions are shown in Figure 11. For all mixtures, no significant improvement in flexural strength was observed with the addition of fibers or SPs.

Generally, the inclusion of PVA fibers is known to suppress microcrack propagation, thereby enhancing the flexural strength and toughness of cementitious composites [35,36,37,46,47]. However, in the present study, this reinforcing effect was not clearly observed. At 56 days, the mortar without fibers exhibited higher strength, and no consistent trend with respect to fiber content was evident at other curing ages.

This suggests that the PVA fiber content used in this study—relative to the fiber geometry and the adopted mix conditions—was insufficient to fully develop the reinforcing effect. Therefore, evaluating fiber contribution based solely on peak flexural strength is difficult. To examine fiber effects in greater detail, load and mid-span deflection during flexural testing were measured using a dynamic strain measurement system (DC-004P, Tokyo Measuring Instruments Laboratory Co., Ltd., Shinagawa-ku, Japan).

**Figure 12:**
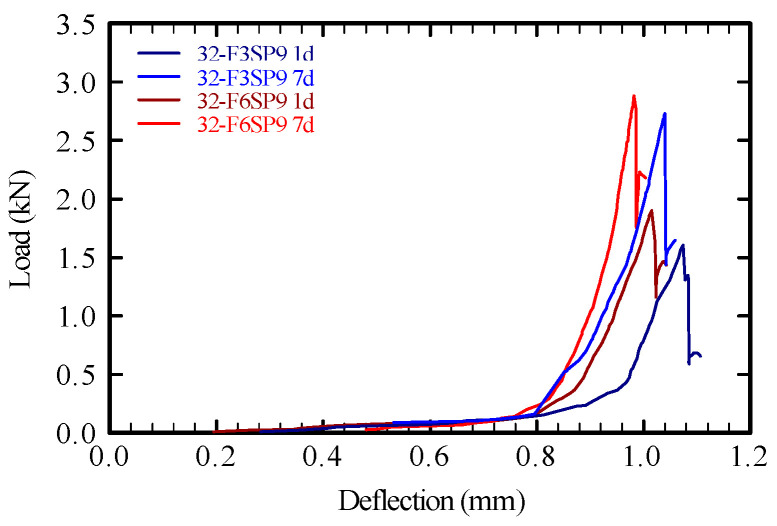
Load–deflection at the specimen center.

The relationship between load and mid-span deflection during the flexural test is depicted in Figure 12. To isolate the effect of fiber content, mixtures 32-F3SP9 and 32-F6SP9 were compared at curing ages of 1 and 7 d.

At both curing ages, the specimen with 0.6% fiber content exhibited smaller deflection at failure than the specimen with 0.3%, indicating a slight improvement in toughness with increasing fiber content and curing time.

In all specimens, the load dropped immediately after reaching the maximum value due to cracking, followed by a slight rebound. At 1 d of age, specimens with 0.6% fiber content tended to show a smaller load drop than those with 0.3% fiber content. Thus, the fibers provided limited resistance to bending stress during early strength development. However, at a fiber content of ≤0.6%, the flexural strength did not improve significantly.

Therefore, in this system, the PVA fibers primarily enhanced internal structural build-up and shape retention in the fresh mortar. These effects facilitated the formation, retention, and reconstruction of internal structure and consequently enhanced the shape stability of layered elements. During early hardening, the fibers also appeared to suppress post-cracking stress reduction and contribute to the initial stability of the layered structure.

The load–deflection curves (Figure 12) indicate that PVA fibers influenced the post-cracking behavior. However, the toughness-related parameters, such as fracture energy, toughness indices, or residual strength, could not be quantified in this study. This represents a key limitation of the mechanical evaluation. Because the primary focus of this work was to establish a practical method for assessing the rheological properties of 3DP mortars, a detailed toughness assessment was beyond the intended scope.

Nevertheless, the limited improvement in flexural strength observed in this study may be attributed not only to the relatively low fiber content but also to factors such as imperfect fiber dispersion, interfacial bonding characteristics, and anisotropy arising from the extrusion-based printing process.

### 3.6. Validity and Applicability of the Vane Shear Test in 3DP Layering Considering Fiber Orientation and Anisotropy

#### 3.6.1. Relationship Between Maximum Shear Stress and Interlayer Stability

**Figure 13:**
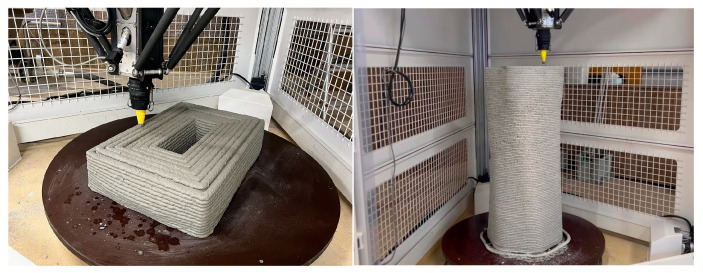
Results of layering test implementation.

The results of the layering tests for the mixtures marked with an asterisk (*) in Table 2 are presented in Figure 13. Mixture 32-F3SP9 exhibited a maximum shear stress of 2.39 kN/m^2^, exceeding the lower control limit of 2 kN/m^2^, demonstrating stable interlayer support during deposition. In contrast, mixture 33-F3SP9, with a maximum shear stress of 0.91 kN/m^2^, failed to support subsequent layers and was therefore unsuitable for stable layering.

This critical threshold is directly linked to the printing process. When the yield stress falls below this level, a deposited strand cannot support the subsequent layer, resulting in interlayer collapse; conversely, excessively high yield stress can cause extrusion instability or nozzle blockage. Thus, approximately 2 kN/m^2^ defines a practical window for the allowable interlayer time interval and the maximum self-supporting build height in our system.

In this study, interlayer stability was assessed through the self-supporting behavior observed during printing, consistent with the primary objective of establishing a material-based indicator of early buildability. Specimens for compressive and flexural strength tests were extracted from the layered structure of mixture 32-F3SP9 after printing.

#### 3.6.2. Compressive Strength of Printed Elements

**Figure 14:**
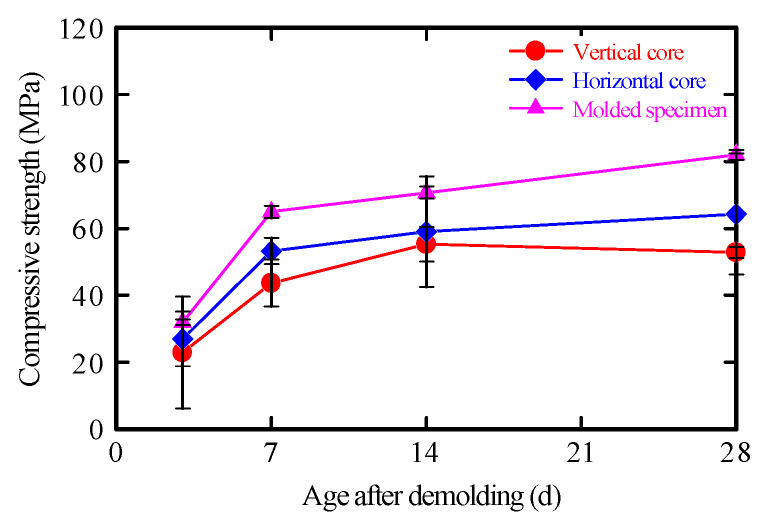
Compressive strength of layered testing.

The compressive strengths of cores extracted in the vertical and horizontal directions from the layered specimen are shown in Figure 14. Core extraction was performed at a curing age of 3 days. For comparison, the strengths of molded specimens made from the same mortar, cast without layering and cured on site, are also presented. At all measured curing ages, the molded specimens exhibited the highest compressive strength. At 28 days, the strength reduction was approximately 22% and 36% for vertical and horizontal cores, respectively. This difference is attributed to the orientation between the loading direction and the layer interfaces [60]. Vertical cores are structurally advantageous because the load is applied parallel to the layer-stacking direction, reducing the likelihood of crack initiation across interfaces. In contrast, horizontal cores are more susceptible to delamination because the load is applied perpendicular to the interfaces, promoting interfacial cracking and reducing compressive strength. Therefore, these results clearly reflect the anisotropy inherent to the layered structure in extrusion-based 3DP mortars.

#### 3.6.3. Flexural Strength of Printed Elements

**Figure 15:**
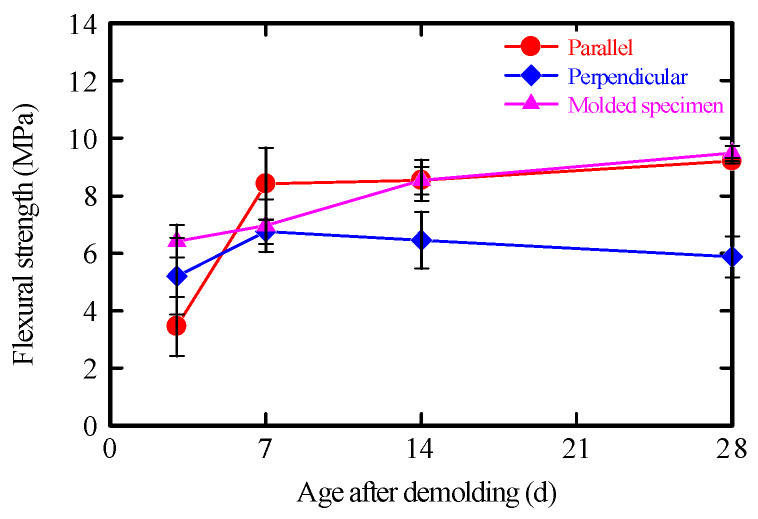
Flexural strength of layered testing.

The flexural strengths of prismatic specimens taken from the laminated structure in both the printing direction and the direction perpendicular to printing are shown in Figure 15. For comparison, prismatic specimens cast in molds using the same mortar were also tested. In the printing direction, the specimens exhibited flexural strength comparable to that of the molded specimens at 28 days.

Previous studies have shown that 3DP materials exhibit anisotropic mechanical properties depending on printing parameters and loading orientation [40], and that fiber orientation does not always result in increased peak flexural strength. In the present study, interlayer discontinuities and microdamage introduced during the specimen extraction process may have limited observable strength enhancement.

In contrast, in the direction perpendicular to printing, cracking occurred predominantly along the layer interfaces, resulting in lower failure loads than in the printing direction. Comparison with the molded specimens indicates that these differences in flexural strength are primarily attributable to material anisotropy induced by the interlayer interfaces.

These results suggest that mixture design for 3DP mortars must account for strength reductions associated with layer interfaces and anisotropic behavior in printed elements.

However, this study presents several limitations. Only a single fiber type was examined, and the effects of fiber length, geometry, and dispersibility on mechanical performance were not fully investigated. Moreover, the discussion on fiber orientation effects was limited to inferences based on mechanical behavior, as the internal orientation state was not directly evaluated. In addition, interlayer adhesion during printing and the long-term durability of printed materials require further investigation.

## 4. Conclusions

This study established a method for evaluating static yield stress and early structural build-up of 3DP mortars under practical construction conditions. To verify the applicability of parameters obtained from laboratory tests, layer-by-layer printing experiments were conducted using an actual 3DP system. The main findings are summarized as follows:

1. Vane shear test: The bridging effect of PVA fibers increased the resistance to shear under stirred conditions, resulting in higher shear stress compared to the static condition. In fiber-reinforced mortars, excessive stirring may reduce flowability.
2. 15-stroke flow test: A pronounced decrease in flow was observed under stirred conditions, attributed to fiber re-agglomeration and a transition to a more cohesive flow behavior. Therefore, this test may be unsuitable for evaluating the flowability of fiber-reinforced mortars.
3. Correlation analysis: The relationship between the maximum shear stress ratio and the 15-stroke flow ratio confirmed that internal structural changes induced by fiber addition affect flowability. The vane shear test can sensitively detect these changes, making it an effective method for evaluating static yield stress and structural build-up of 3DP mortars.
4. Compressive strength: The highest compressive strength was achieved at a fiber content of 0.3%, while excessive fiber addition led to strength reduction due to decreased density and fiber agglomeration. Additionally, an excessive dosage of the superplasticizer weakened interfacial bonding, hindering strength development.
5. Flexural strength: Although fiber addition did not lead to a clear improvement in strength or toughness, the resulting load–displacement response showed less deflection at failure for a fiber content of 0.6% than for a content of 0.3%, as well as a lower early-age load reduction at a curing age of 1 day.
6. Interlayer stability: During layering tests using an actual 3DP system, mortars with a maximum shear stress below 2 kN/m^2^ were unable to support themselves and were difficult to layer. This demonstrates that the vane shear test is effective for predicting interlayer stability.
7. Compressive strength of printed elements: The compressive strength of cores extracted from the laminated structure decreased both vertically and horizontally due to the influence of layer interfaces, with a more pronounced reduction in the horizontal direction.
8. Flexural strength of printed elements: The flexural strength the laminated structure in the printing direction was comparable to that of molded specimens, and the contribution of fiber orientation was not evident at peak strength. In contrast, strength in the direction perpendicular to printing decreased markedly, demonstrating clear interlaminar anisotropy.

The findings of this study provide quantitative indicators for assessing the effects of fiber addition and superplasticizer dosage on mixture and construction management for 3DP mortars. In particular, the vane shear test offers a practical and effective approach for onsite quality control as it sensitively detects changes in internal structure and static yield stress development induced by fibers. Because fiber content and admixture dosage substantially affect both printability and mechanical performance, these parameters should be treated as key factors in mixture proportioning and field operations. In conclusion, vane shear testing can be used for optimizing print performance in real-time scenarios.

Future research should be focused on systematic evaluations of the effects of fiber length, geometry, and dispersibility on structural build-up and mechanical performance. The direct measurements of internal fiber orientation are required to clarify its contribution to anisotropy in printed elements.

In addition, direct mechanical tests of interlayer bond strength, such as splitting tensile or direct shear tests on bilayer printed specimens, should be incorporated to obtain a quantitative engineering parameter for structural design. These measurements will enable a more rigorous evaluation of interlayer performance and allow for correlation with the static yield stress obtained from the vane shear test.

The long-term durability of printed materials—including shrinkage, microcracking, and environmental resistance—should be investigated to establish robust design methodologies and enhance the practical applicability of 3D-printed mortars.

Finally, future research should incorporate quantitative toughness measurements—such as fracture energy, toughness indices, and post-crack residual strength—to more clearly elucidate the reinforcing mechanisms of PVA fibers and their contribution to post-crack behavior. Such measurements will complement the framework established in this study.

## Figures and Tables

**Table 1:** Raw material specifications.

Material	Abbreviation	Details
Water	W	Tap water (Density: 1.00 g/cm^3^)
Cement	BB	Blast-furnace cement Type B (Density: 3.04 g/cm^3^; Brain: 3770 cm^2^/g)
Reactive filler	SF	Silica fume (Density: 2.29 g/cm^3^; BET: 184,000 cm^2^/g)
Inert filler	Ca	Calcium carbonate powder (Density: 2.60 g/cm^3^; BET: 14,900 cm^2^/g)
Fine aggregate	S	Silica sand No. 4 (Place of origin: Nankan, Tamana, Japan; Density: 2.60 g/cm^3^; Particle size distribution: 0.212–1.18 mm)
Admixture	SP	Superplasticizer Type I (Density: 1.20 g/cm^3^)
Fiber	F	Polyvinyl alcohol (PVA) fibers (Density: 1.30 g/cm^3^; Length: 6 mm; Diameter: 40 µm; Elastic modulus: 23–40 GPa)

**Table 2:** 3DP mortar mix proportions.

Name	W/B (%)	S/P (%)	(kg/m^3^)						
			W	P			S	F (Vol.% of Mortar)	SP (Mass% of Water)
				B		Ca			
				BB	SF				
32-F0SP9	32	49	320	928	86	254	621	0 (0)	9.1 (0.9)
32-F3SP9 *								3.9 (0.3)	
32-F6SP9								7.8 (0.6)	
32-F0SP11								0 (0)	11.2 (1.1)
32-F3SP11								3.9 (0.3)	
32-F6SP11								7.8 (0.6)	
33-F0SP9 *	33	52	330	910	90	223	636	0 (0)	9.0 (0.9)

**Table 3:** Specifications of the vane blade.

Symbol	Diameter (mm)	Height (mm)
A	10	20
B	15	30
C	20	40
D	30	60

## Data Availability

The original contributions presented in this study are included in the article. Further inquiries can be directed to the corresponding author.

## Abbreviations

The following abbreviations are used in this manuscript:

3DP	Three-dimensional printing
PVA	Polyvinyl alcohol
W	Water
BB	Blast furnace slag cement type B
SF	Silica fume
B	Binder
Ca	Calcium carbonate
S	Sand
SP	Superplasticizer
F	Fiber
BET	Brunauer–Emmett–Teller specific surface area
W/B	Water-to-binder ratio (Binder = BB + SF)
S/P	Sand-to-powder ratio (Binder = BB + SF + Ca)
